# Prediction of irritable bowel syndrome by integrating urine metabolites and gut microbiota

**DOI:** 10.1038/s41598-025-22068-1

**Published:** 2025-10-31

**Authors:** Gayoun Lee, Mee-Hyun Lee, Seong-Eun Park, Juhan Pak, Soobin Bae, Suryang Kwak, Young-Ho Moon, Hong-Seok Son

**Affiliations:** 1 Department of Biotechnology College of Life Sciences and Biotechnology , Korea University , 02841 Seoul, Republic of Korea; 2 College of Korean Medicine , Dongshin University , 58245 Naju, Republic of Korea; 3 Department of Bio and Fermentation Convergence Technology, College of Science and Technology, Kookmin University, 02707 Seoul, Republic of Korea; 4 Mokpo Korean Medicine Hospital, Dongshin University , 58665 Mokpo, Republic of Korea

**Keywords:** Irritable bowel syndrome, Gut microbiota, Urinary metabolites, Metabolomics, Next-generation sequencing, Predictive markers, Gastrointestinal diseases, Dysbiosis

## Abstract

Irritable bowel syndrome (IBS) is a functional gastrointestinal disorder characterized by recurrent abdominal pain and altered bowel habits. While gut microbiota alterations and metabolic disturbances have been implicated in IBS, their potential role in classification and patient stratification remains unclear. This study aimed to evaluate the potential of integrating gut microbiota profiling and urinary metabolomics for improved IBS classification. Fifty-four participants (27 healthy controls, 27 IBS patients) were recruited for gut microbiota and urinary metabolite analysis. Gut microbiota composition was assessed via 16S rRNA gene sequencing, and urinary metabolites were profiled using gas chromatography-mass spectrometry (GC-MS). Receiver operating characteristic (ROC) curve analysis was performed to assess the predictive accuracy of gut microbiota, urinary metabolites, and their combined model. LEfSe analysis identified that Clostridia and Prevotella as enriched in IBS patients, whereas Bacteroidales and Faecalitalea were predominant in healthy individuals. Urinary metabolite analysis revealed significant alterations in metabolite profiles, with IBS patients exhibiting elevated fructose levels and trends of increased serine, mannose, and galactose. ROC curve analysis demonstrated that urinary metabolomics (AUC = 0.65) outperformed gut microbiota profiling (AUC = 0.54), while a combined approach integrating both datasets achieved the highest predictive accuracy (AUC = 0.74). These findings indicate that integrating urinary metabolomics with gut microbiota profiling may provide preliminary insights into IBS classification. Given the small sample size, potential risk of overfitting, absence of external validation, and possible dietary or medication confounding, the observed performance of the combined approach should be regarded as hypothesis-generating rather than confirmatory. Nevertheless, the results highlight the potential utility of integrative omics strategies in IBS research, underscoring the need for validation in larger, sex-balanced, and clinically diverse cohorts to establish their robustness and broader relevance.

**Supplementary Information:**

The online version contains supplementary material available at 10.1038/s41598-025-22068-1.

## Introduction

Irritable bowel syndrome (IBS) is a chronic functional bowel disorder characterized by recurrent abdominal pain and altered bowel habits, including changes in frequency and consistency, often associated with defecation^1,2^. It presents with a range of symptoms, including abdominal discomfort, pain, urgency during bowel movements, bloating, diarrhea (IBS-D), and constipation (IBS-C), significantly impacting patients’ quality of life^3^. A systematic review of IBS prevalence across various global studies found that approximately 10% of the population is affected by IBS​^4^. The prevalence of IBS varies widely across countries, ranging from 1.1% in France and Iran to 35.5% in Mexico, with similarly uneven distribution observed across Asian nations^5^. These differences may be attributed to various factors such as differences in diagnostic criteria, lifestyle, diet, healthcare access, and regional variations in genetic and environmental influences^6^. Among these contributing factors to IBS, gut microbiota alterations have gained increasing attention as a key factor in its pathophysiology^7^. Specifically, disruptions in the gut microbial community can influence intestinal motility, immune function, and gut permeability, which may contribute to IBS symptoms via dysregulation of the brain–gut axis^8^.

Building on this evidence, recent studies have further investigated gut microbiota alterations in IBS and their potential role in disease development. Studies have identified significant alterations in the gut microbiota of IBS patients, including reduced microbial diversity and shifts in specific bacterial populations, such as increased Firmicutes and Proteobacteria, along with decreased *Lactobacillus*, *Bifidobacterium*, and *Faecalibacterium prausnitzii*^9,10^. These microbial imbalances have been linked to dysregulated gut motility, immune activation, and altered fermentation patterns, potentially leading to common IBS symptoms like bloating and abdominal pain^11^. However, the relationship between IBS and gut microbiota remains complex and inconsistent across studies. While some research has identified specific microbial signatures in IBS patients, other studies have reported discordant findings, with significant variability in microbial composition among individuals and across different populations^12^. Due to high inter-individual variability in gut microbiota, no single microbial marker reliably distinguishes IBS, making diagnosis based solely on microbial composition challenging^8^.

As microbiota-based prediction remains challenging, urine metabolomics has gained significant attention recently due to its non-invasive collection method and its ability to provide comprehensive metabolic information^13^. Urine is a biofluid composed of water, electrolytes, and a diverse range of metabolic by-products excreted by the kidneys, reflecting systemic physiological and pathological conditions^14^. It contains a wide array of metabolites, making it a valuable biofluid for disease diagnosis and monitoring^14^. Additionally, urine is relatively stable and free from interfering proteins or lipids, facilitating accurate metabolomic studies^15^. These properties make urine metabolomics a powerful tool for identifying potential biomarkers for various diseases. Recent studies indicate that urinary metabolomics effectively distinguishes inflammatory bowel disease, gastrointestinal cancers, and celiac disease from healthy controls by identifying key metabolic alterations^16,17^. Also, a strong connection has been identified between urinary metabolites and gut microbiota with specific microbes influencing amino acid metabolism, energy production, and microbial co-metabolism^18^. As a non-invasive approach, urinary metabolic profiling captures gut microbiota-associated changes, revealing microbial-derived metabolites that reflect host-microbiome interactions and systemic metabolic shifts^19^. Despite these promising findings, research on IBS biomarkers through urine metabolite analysis remains limited, necessitating further studies to establish reliable non-invasive diagnostic markers for IBS.

Therefore, this study aims to comprehensively analyze and compare intestinal microbiota and urinary metabolite profiles of healthy individuals and IBS patients to identify potential diagnostic biomarkers for IBS. This study could provide insights into the microbial and urinary metabolic alterations associated with IBS, contributing to a better understanding of its pathophysiology and development of integrated metabolomic and microbiota-based diagnostic strategies.

## Materials and methods

### Study cohort

 This study was approved by the Ethics Committee of Dongshin University Korean Medicine Hospital (NJ-IRB-23-1) in March 2023 and conducted in accordance with the principles of the Declaration of Helsinki. A total of 54 participants were enrolled in this study, comprising 27 healthy controls and 27 IBS patients. Recruitment was conducted at the Korean medicine hospital of Dongshin university (Mokpo, Jeonnam, Republic of Korea), and through local advertisements. All participants provided written informed consent before joining the study. The age range of participants was 21–62 years. To reduce clinical heterogeneity and focus on a more defined disease subtype, the IBS cohort was restricted to patients meeting the Rome IV diagnostic criteria for diarrhea-predominant IBS (IBS-D), as defined by Drossman et al.^20^. They also completed validated self-assessment questionnaires to measure the severity of their IBS symptoms, as shown in **Table ****S1**. The control group consisted of individuals who had no history of IBS or other gastrointestinal diseases and displayed normal clinical health based on screening. Exclusion criteria included the presence of severe conditions such as cancer or gastrointestinal disorders unrelated to IBS (e.g., Crohn’s disease, gastrointestinal malignancy) that could explain their symptoms, as well as other factors deemed inappropriate by the investigators. Participant characteristics are presented in **Table S2.**

### Sample collection

Fecal and urine samples were collected from all participants. Fecal samples were self-collected at home using NBgene tubes (Noble Biosciences). Following an overnight fast, urine samples were freshly collected midstream in the morning at the hospital from each participant, using sterile 50 mL conical tubes. All samples were transported to the laboratory in mini cooler bags containing ice packs and subsequently stored at − 80˚C until analysis.

### 16S rRNA gene sequencing and data processing

DNA extraction from the fecal samples was performed using the AccuFAST automation system (AccuGene Inc., Incheon, Republic of Korea) following the manufacturer’s instructions. For MiSeq sequencing, bacterial genomic DNA was amplified using primers with 515 forward and 806 reverse bases, incorporating Unique Dual (UD) index adapter sequences to target the V4 hypervariable region of the 16S rRNA genes. Amplification of the 16S rRNA genes was performed through 25 cycles of polymerase chain reaction (PCR) using KAPA HiFi HotStart ReadyMix (Roche sequencing, USA). The resulting PCR products (~ 250 bp) were purified with HiAccuBeads for next-generation sequencing (NGS) library preparation (AccuGene Inc.). The amplicon libraries were then pooled at equimolar concentrations and sequenced on an Illumina MiSeq platform using the MiSeq Reagent Kit v2 for 500 cycles (Illumina, USA).

 All reads were denoised by correcting amplicon errors and used to infer exact amplicon sequence variants (ASVs) using DADA2 v1.16^21^. The final library sizes ranged from 44,508 to 150,242 reads per sample, with a mean of 116,812 reads across all samples. Taxonomic classification at all hierarchical levels was conducted using the QIIME 2 framework^22^, utilizing a Naïve Bayes classifier trained on the SILVA 138.1 rRNA reference database for taxonomic assignments^23^. For α- and β-diversity analyses, sequencing depth was rarefied to 20,000 reads per sample, and taxonomic relative abundance tables were generated from normalized counts. The reporting of microbiome data in this study follows the STORMS (Strengthening The Organization and Reporting of Microbiome Studies) guidelines, and the completed checklist is provided in **Table S3**^24^.

### Gas chromatography-mass spectrometry (GC-MS) analysis and data processing

The metabolic analysis protocol and GC-MS conditions were based on those reported in a previous metabolomic study^25^, with slight modifications. The urine samples were gradually thawed on ice before analysis. 100 µL of urine samples were mixed with 900 µL of methanol. After mixing thoroughly for 5 min, the samples were centrifuged at 15,928$\:\:\times\:$ g for 10 min at 4˚C. 400 µL of the supernatant was then combined with 20 µL of a ribitol solution (internal standard, 0.5 mg/mL in water) and then the samples were subjected to centrifugal vacuum concentrator (VC2124, Hanil Scientific Inc., Gimpo, Republic of Korea) for 12 h. To evaluate the stability and reliability of the analysis, quality control (QC) samples were prepared by blending 30 µL from each sample. After vacuum drying, samples were then treated with 100 µL of O-methoxyamine hydrochloride (20 mg/mL in pyridine) and ultrasonicated using a Powersonic 520 (Hwashin, Seoul, Republic of Korea) at 4˚C for 20 min. Following a 1.5minute mixing period, the samples were incubated in the dark at 30˚C with continuous shaking at 75 rpm for 90 min. The samples were subsequently silylated with 50 µL of N-methyl-N-(trimethylsilyl) trifluoroacetamide (MSTFA) and incubated with shaking at 75 rpm and 37˚C for 30 min. Following the incubation, the samples were centrifuged at 13,572$\:\:\times\:$ g for 5 min at 4˚C, and 80 µL of the supernatant was transferred into a 2 mL glass vial equipped with a 100 µL insert for metabolite analysis.

The derivatized samples were analyzed by GC–MS (QP2020, Shimadzu, Kyoto, Japan) with an RTX-5MS capillary column (Restek, Bellefonte, PA, USA). GC–MS conditions were as follows: injector temperature at 230˚C; transfer line temperature at 250˚C; helium flow rate of 1 mL/min, detector at 280˚C. The GC oven temperature program began at 80˚C, held for 2 min, then increased at 15˚C/min to a final temperature of 330˚C, held for 6 min. To ensure stability, performance, and reproducibility, QC samples were analyzed alongside the test samples. Specifically, separate pooled QC and blank sample (BS) sets were prepared and injected at the beginning of the batch prior to the study samples, and additional sets were injected after every 20 study samples throughout the analytical run to monitor instrument stability and potential drift.

 The retention index (RI) values of the compounds were calculated using the retention times (RT) of C7–C40 alkane standards (Sigma-Aldrich, St. Louis, MO, USA) under identical GC–MS conditions. Raw peak data were processed and converted to ABF files using Shimadzu GC–MS Postrun Analysis (Shimadzu) and MS-DIAL v. 4.9. Identification was performed with a retention index tolerance of 20, an EI similarity cut-off of 90%, and an identification score cut-off of 90%, referencing EI-MS and Kovats RI. The identified spectra were further matched to the NIST v. 20.0 library and standard reagents. Peak intensities were normalized to ribitol, and subsequently normalized by total peak area (TPA, sum normalization) to account for differences in overall urine concentration. To improve data quality, features with an average intensity < 1 in QC or blank samples were excluded, and metabolites with poor reproducibility (QC %RSD > 30%) across pooled QC samples were removed prior to statistical analysis. The reporting of metabolomics data adheres to the MSI (Metabolomics Standards Initiative) recommendations, with the corresponding checklist summarized in **Table S4**^26^.

### Statistical analyses of the omics data

Statistical analyses and data visualizations were performed in R version 4.3.3. Diversity metrics were calculated using vegan^27^ and ape^28^ packages. Permutational multivariate analysis of variance (PERMANOVA) was performed with adonis2 in the vegan package^27^ to evaluate the structural distinctiveness of gut microbiota and urinary metabolites between the healthy and IBS groups. This analysis was performed via principal coordinates analysis (PCoA) based on both Bray-Curtis and Jaccard indices. Taxonomic features specifically associated with healthy or IBS groups were identified using linear discriminant analysis effect size (LEfSe)^29^. To minimize false positives due to the compositional nature of microbiome data, we additionally performed compositional differential abundance analysis using the Analysis of Compositions of Microbiomes with Bias Correction (ANCOM-BC) method across all taxonomic ranks. A Kruskal–Wallis test was used for group comparisons, and false discovery rate (FDR) correction was applied to control for multiple testing. Taxa with FDR-adjusted *q*-values < 0.05 and logarithmic linear discriminant analysis (LDA) scores > 2.5 were considered significant. This FDR correction was explicitly applied to microbiota analyses to minimize the risk of false positives in high-dimensional comparisons. Receiver operating characteristic (ROC) curves were generated using the roc function in the pROC package^30^ to evaluate the diagnostic accuracy of identified taxonomic and metabolomic features. To construct multivariable ROC models under the constraint of events-per-variable (EPV ≥ 5), the number of predictors was limited to five per model. Specifically, for the combined model integrating gut microbiota and urinary metabolite data, the top three urinary metabolites and top two microbial taxa were selected based on their univariate AUC rankings. This ratio was determined to balance representation from both omics layers while complying with the total predictor limit, allowing sufficient diversity without overfitting. To enable integrative modeling of gut microbiota and urinary metabolite data, all features were standardized using Z-score normalization. Diagnostic performances of models were quantified and evaluated based on corresponding area under the curve (AUC). To reduce the risk of overfitting, all ROC analyses were conducted using 5-fold cross-validation with ridge logistic regression (α = 0). The AUC values were averaged across folds, and 95% confidence intervals were calculated using the pROC package. All analysis results were visualized using ggplot2 package^31^.

Additional statistical analyses for gut microbiota and urinary metabolite data were performed using GraphPad Prism software (version 9.4.1, GraphPad Software Inc., San Diego, California, USA). Normality of the data was evaluated using the Shapiro–Wilk test. For variables following a normal distribution, Welch’s t-test was applied; for non-normally distributed variables, the Mann–Whitney U test was used. To account for multiple comparisons, raw *p*-values from these tests were adjusted using the Benjamini–Hochberg FDR procedure for both microbial taxa and urinary metabolites. Urine data variance was visualized using partial least squares discriminant analysis (PLS-DA) score plots of GC-MS data generated with SIMCA-P 17.0 (Umetrics, Umea, Sweden). The quality of the model score plot was assessed using *R*^*2*^ (variance in the data) and *Q*^*2*^ (prediction of the model) values. Results are reported as mean ± standard deviation, with statistical significance set at *p* < 0.05. Baseline demographic variables such as age, body mass index, and sex were compared between the two groups. Independent t-tests were used for the continuous variables age and body mass index, and the chi-squared test was applied for the categorical variable sex. The results are presented in **Table S2**. For univariate metabolite analysis, multiple-testing correction was performed using the FDR method, and only metabolites with *q*-values < 0.05 were considered significant. Metabolite set enrichment analysis (MSEA) was conducted using MetaboAnalyst 6.0 (https://www.metaboanalyst.ca; accessed on 6 Nov 2024).

## Results and discussion

### Clinical characterization of IBS patients

 All IBS patients enrolled in this study met the Rome IV diagnostic criteria and were classified as having IBS-D, based on both clinical diagnosis and questionnaire responses as shown in **Table S5**. Symptom severity scores further supported the clinical characterization of this cohort. The mean abdominal pain severity score was 53.96 ± 12.34, and abdominal pain was reported on an average of 4.07 ± 1.57 days over the past 10 days. The mean bloating severity score was 50.75 ± 16.69. In addition, all patients reported an average maximum pain score of ≥ 3.0 on a 0–10 scale, and the number of days with at least one loose stool (Bristol stool type 6 or 7) was 3.85 ± 1.51. These findings indicate that the enrolled IBS-D patients experienced clinically meaningful abdominal symptoms, consistent with moderate-to-severe symptom burden, thereby highlighting the importance of integrating symptom-based assessments with microbiota and metabolite profiling in this population.

### Taxonomic composition and diversity assessment of gut microbiota

**Fig. 1:**
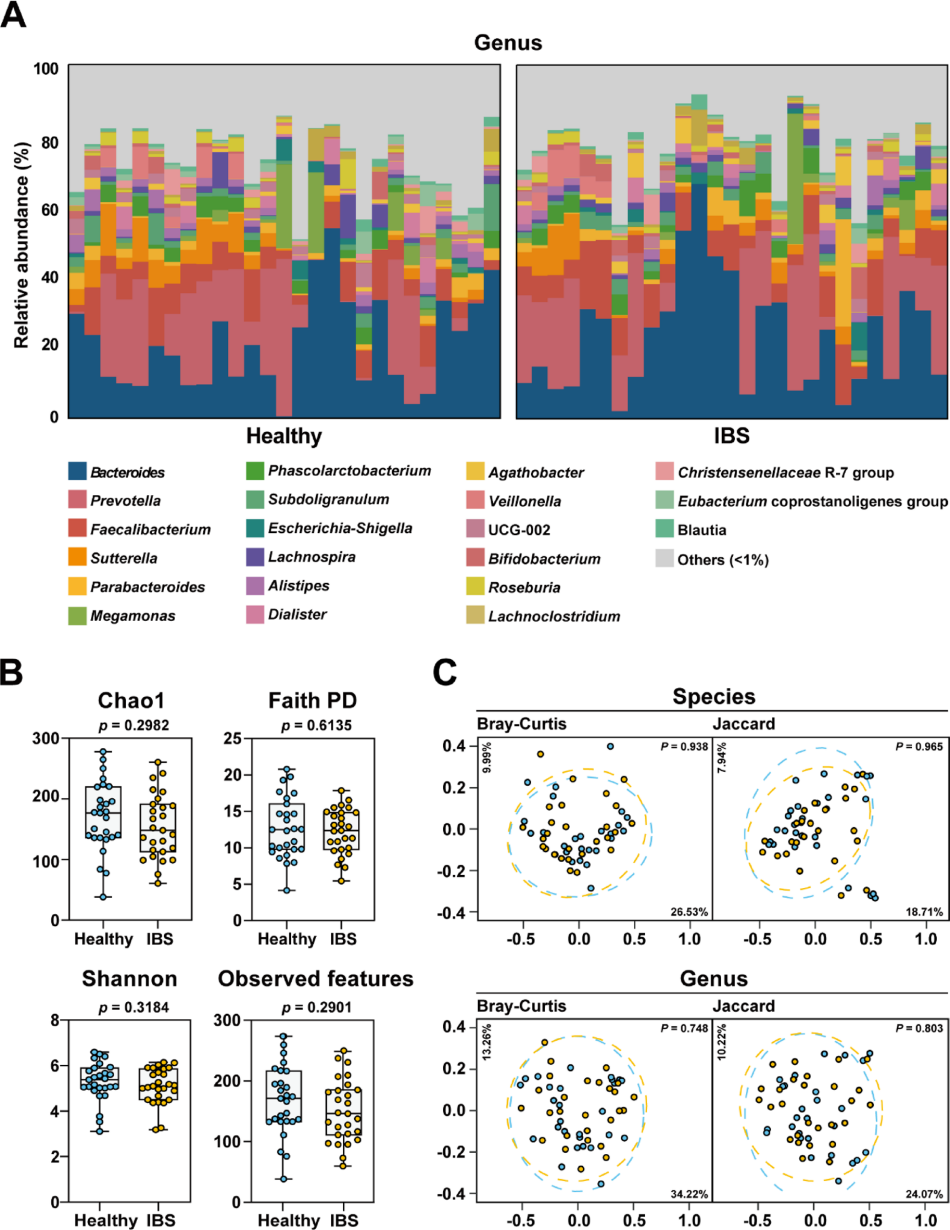
Comparative analysis of gut microbiota taxonomic structures in healthy and IBS groups. (**A**) Bar charts depicting the relative abundance (%) of microbiota at the genus level in the healthy and IBS groups. Colors represent bacterial genera, as indicated in the legend below the panel; genera with an average abundance of less than 1% are grouped as ‘Others (< 1%)’. (**B**) α-diversity analysis comparing the healthy and IBS groups, based on Chao1, Faith’s Phylogenetic Diversity (PD), Shannon index, and Observed features. Data are shown as box plots with whiskers representing mean ± SD. (**C**) Principal coordinate analyses of the taxonomic structures of the gut microbiota at species and genus levels of healthy and IBS groups, based on Bray-Curtis and Jaccard indices. *P-*values for group comparisons were computed via permutational analysis of variance (PERMANOVA).

We investigated the taxonomic composition of gut microbiota to evaluate its potential in differentiating individuals with IBS from healthy controls. As shown in Fig. 1A, We investigated the taxonomic composition of gut microbiota to evaluate its potential in differentiating individuals with IBS from healthy controls. As shown in Fig. 1A, the genus level microbiota composition exhibited individual variability, but showed no clear distinction between the two groups. Dominant genera such as *Bacteroides*, *Prevotella*, and *Faecalibacterium* were consistently present in both cohorts, suggesting that broad taxonomic shifts may not be the main factor driving IBS-related microbial changes.

To further assess the microbiota structure, we analyzed α-diversity, which reflects species richness and community evenness within individuals (Fig. 1B). Diversity metrics, including Chao1, Faith’s Phylogenetic Diversity (PD), Shannon index, and Observed feature, showed no significant differences between the healthy and IBS groups. These findings indicate that overall microbial diversity remains comparable between groups.

 To examine the broader structural differences between groups, we performed β-diversity analysis using Bray-Curtis and Jaccard indices, visualized through PCoA (Fig. 1C). In both species- and genus-level comparisons, there was a substantial overlap between the healthy and IBS groups, with no statistically significant clustering observed (PERMANOVA, *p* > 0.05 for all comparisons). These results suggest that, at the community level, microbiota differences between healthy individuals and IBS patients may not be pronounced. To complement these community-level analyses, we further conducted univariate statistical testing on the genus-level taxa shown in Fig. 1A, applying Welch’s t-test or Mann–Whitney U test with false discovery rate correction. The corresponding raw *p*-values, FDR-adjusted *q*-values, and effect sizes (Hedges’ *g*) are provided in **Table S6**.

### Differential taxonomic features of gut microbiota

**Fig. 2:**
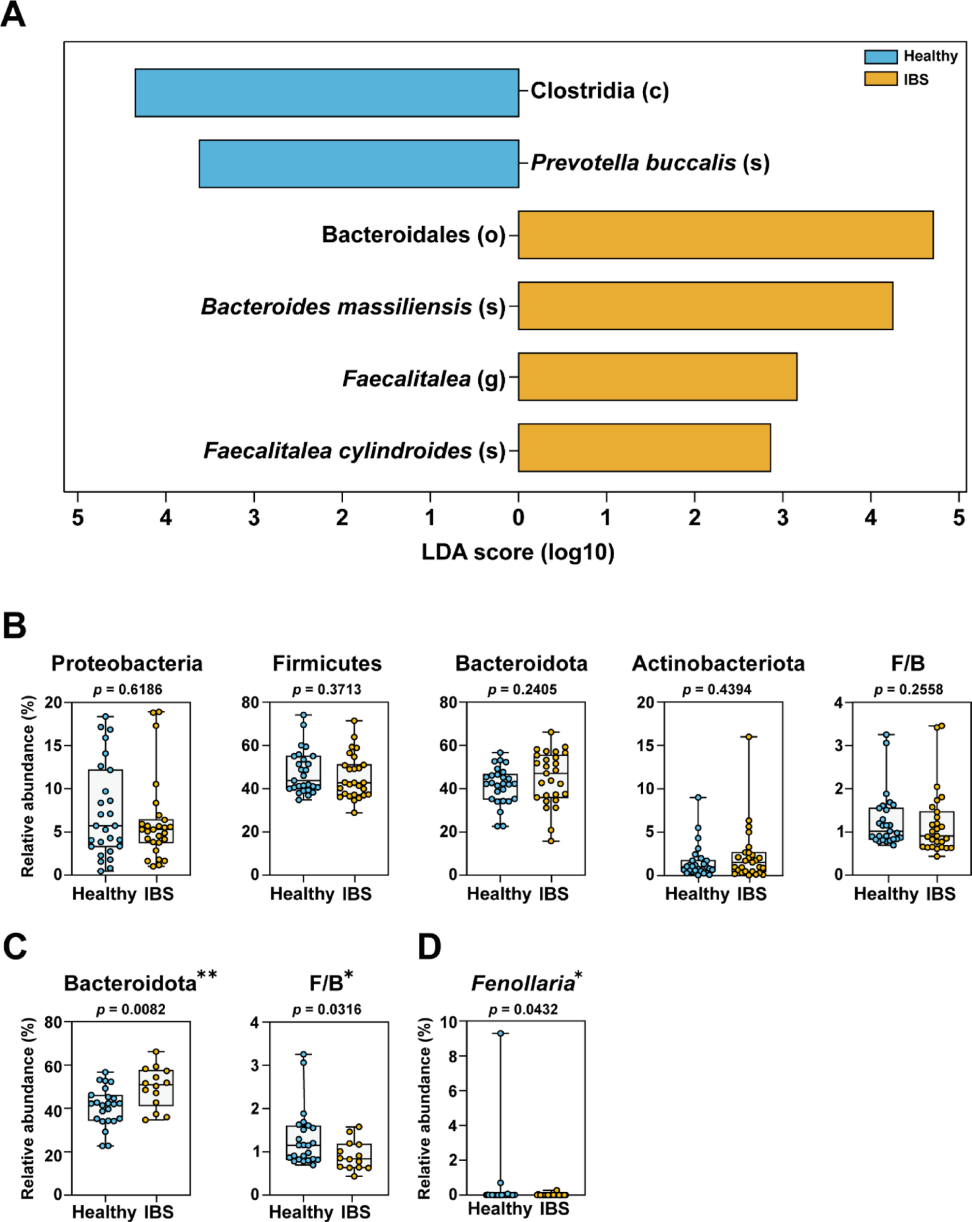
Identification of taxonomic features distinguishing the healthy and IBS groups. (**A**) Taxonomic features identified by LEfSe from gut microbiota 16S rRNA amplicon sequencing data across all taxonomic hierarchy levels. (**B**) Comparisons of relative abundances of identified phylum level features between healthy and IBS groups. (**C**) Box plots showing phylum-level taxa with significant differences (p < 0.05) between healthy and IBS groups in the female subgroup. The 95% CIs of the mean values were: Bacteroidota (Healthy: 37.06–44.56; IBS: 44.25–55.11) and F/B ratio (Healthy: 1.024–1.587; IBS: 0.719–1.106). (**D**) Box plots showing genus-level taxa with significant differences between healthy and IBS groups in the female subgroup. The 95% CIs of the mean values were: Fenollaria (Healthy: –0.3306–1.083; IBS: –0.008734–0.03428). Data are shown as box plots with whiskers representing mean ± SD. Wilcoxon rank-sum tests were performed for group comparisons (*p < 0.05, **p < 0.01).

To identify taxa differentiating healthy and IBS groups, we performed LEfSe across all taxonomic levels of the gut 16S rRNA amplicon sequencing data (Fig. 2A). At higher taxonomic levels, Clostridia was significantly enriched in healthy controls, whereas Bacteroidales and the genus *Faecalitalea* were enriched in IBS. In line with our findings, Zhuang et al.^32^ reported that *Faecalitalea* was significantly increased in patients with IBS-D compared with healthy controls. This enrichment has been interpreted as reflecting a shift toward taxa associated with mucosal inflammation and altered fermentation pathways, which may contribute to symptom generation in IBS-D.

Additionally, we compared the gut microbiota at the phylum level between the overall healthy and IBS groups (Fig. 2B). No significant differences were observed between the two groups at the phylum level, suggesting that broad compositional shifts across major bacterial phyla do not sufficiently distinguish IBS patients from healthy individuals. This finding aligns with previous reports indicating that phylum-level alterations, including changes in the Firmicutes-to-Bacteroidota ratio, are often inconsistent across IBS studies and that microbial dysbiosis in IBS tends to be more pronounced at lower taxonomic levels, such as the genus or species level^9,12^.

It has been reported that individuals with IBS exhibit sex-specific differences in gut microbiota composition^33^. In order to determine whether these differences vary by IBS, we conducted separate analyses for male and female groups, but no significant differences were observed in the male group. At the phylum level, the relative abundance of Bacteroidota and the Firmicutes/Bacteroidota (F/B) ratio differed between healthy and IBS groups in the female group (Fig. 2C). The F/B ratio is a commonly used metric to assess gut microbiota composition. A higher F/B ratio has been associated with conditions such as obesity, while a lower F/B ratio has been linked to inflammatory bowel diseases^34^. Given that findings on the F/B ratio in IBS patients have been inconsistent across studies, our results are presented as exploratory and should be interpreted with caution. At the genus level, *Fenollaria* appeared more abundant in healthy females compared with IBS (Fig. 2D). Lo et al.^35^ reported that *Fenollaria timonensis* was first identified in the gut microbiota of a healthy individual, suggesting its potential association with a balanced gut microbial community. Its ability to produce short-chain fatty acids (SCFAs), which play a crucial role in maintaining gut homeostasis, may potentially contribute to its higher prevalence in healthy individuals. Our observation, however, is exploratory and requires validation in larger, sex-balanced cohorts before any functional implications can be established.

### Analysis of urinary metabolites and metabolic pathways

**Fig. 3:**
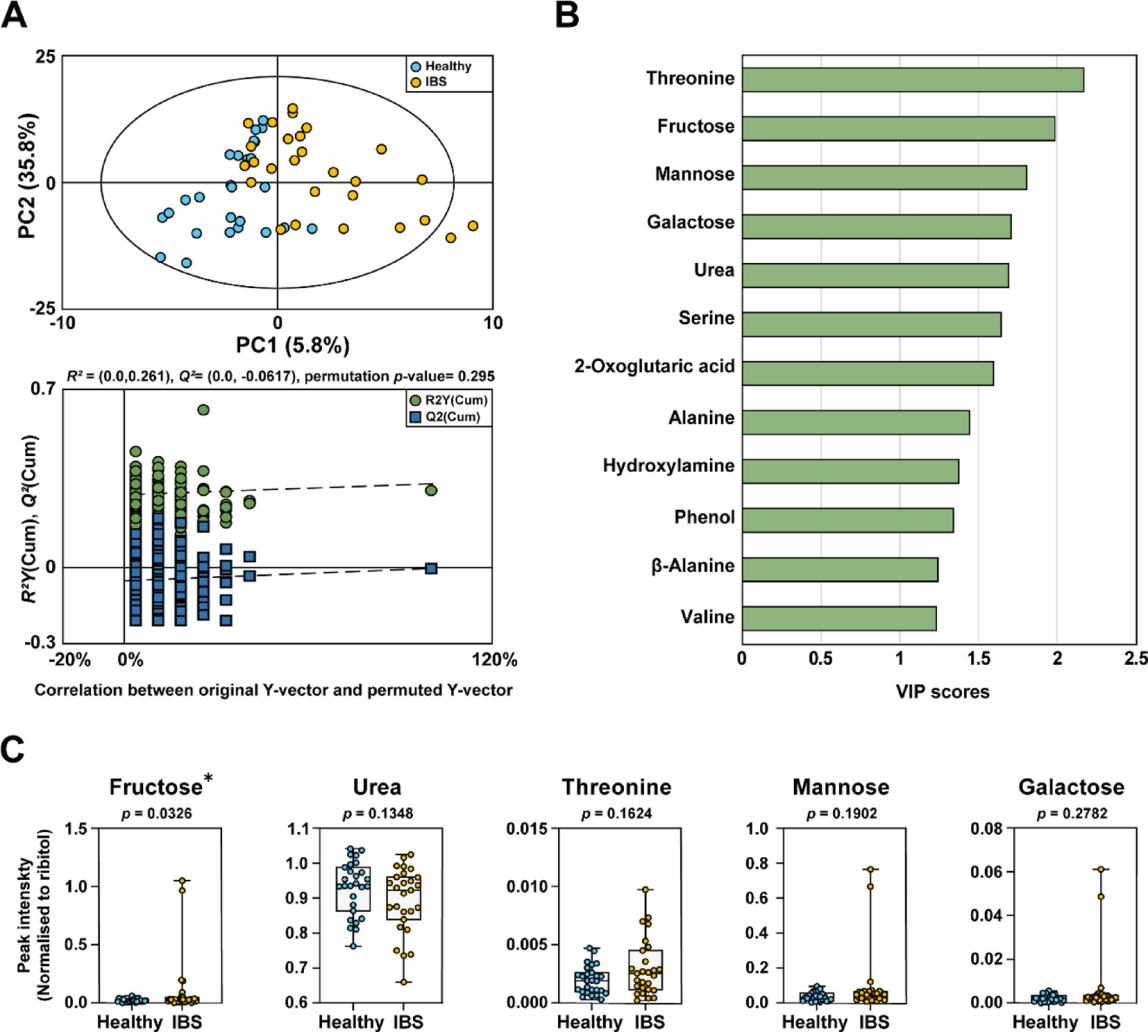
Urine metabolites of the healthy and IBS groups analyzed by GC-MS. (**A**) Projection to latent structures and discriminant analysis (PLS-DA) score plot and 200 permutations test obtained from GC–MS data of urine samples of healthy and IBS groups. (**B**) Identified metabolites with VIP scores (VIP > 1.2), indicating their importance in distinguishing the groups. (**C**) Representative urinary metabolites are shown as box plots with whiskers representing mean ± SD. Group differences were evaluated using Welch’s t-test or Mann–Whitney U test depending on data distribution, with false discovery rate (FDR) correction applied across all tested metabolites.

 To explore metabolic differences between healthy and IBS groups, we conducted a comprehensive analysis of urine metabolites using GC-MS (Fig. 3A-C). To ensure analytical quality, pooled QC samples showed good reproducibility, with a %RSD of 12.05% across all detected metabolites. The PLS-DA score plot demonstrated a clear separation between the healthy and IBS groups, indicating distinct urinary metabolomic profiles between groups. Additionally, a permutation test was performed to evaluate the performance of the PLS-DA model. (Fig. 3A). Metabolites that contributed most significantly to group differentiation were identified (Variable Importance in Projection (VIP) score > 1.2), as shown in Fig. 3B. Twelve metabolites, including threonine, fructose, mannose, galactose, urea, serine, 2-oxoglutaric acid, alanine, hydroxylamine, phenol, β-alanine, and valine, were identified as key contributors to the distinction between the healthy and IBS groups. Among these metabolites, fructose was the only urinary metabolite that showed a significant difference (*p* < 0.05) between the healthy IBS groups (Fig. 3C). Previous studies have suggested that fructose malabsorption is more prevalent in IBS patients, which may explain the increased urinary fructose levels observed in this study^36^. Fructose malabsorption results in unabsorbed fructose reaching the colon, where microbial fermentation produces gas and short-chain fatty acids, exacerbating gastrointestinal symptoms. A study investigating fructose malabsorption in IBS patients revealed that a 25 g fructose hydrogen breath test found a higher prevalence of fructose malabsorption in IBS patients than in asymptomatic controls, supporting its role in IBS pathophysiology^37^. Likewise, urinary metabolite analysis in this study identified fructose as the only significantly elevated metabolite in IBS patients, reinforcing the link between impaired fructose metabolism and IBS. Consistent with previous reports, these findings initially suggested that urinary fructose concentration could serve as a potential biomarker for distinguishing IBS patients from healthy individuals. However, when applying FDR correction across all tested metabolites, this significance was not retained. Nevertheless, fructose stood out as the only metabolite that reached significance at the raw *p*-value level, highlighting its potential as a preliminary candidate biomarker that warrants further validation in future studies. The complete results of univariate testing, including raw *p*-values, FDR-adjusted *q*-values, and effect sizes (Hedges’ *g*), are provided in **Table S7**. In addition, serine tended to be detected at higher levels in the IBS patient group, though the difference was not statistically significant. Elevated urinary serine levels in IBS patients may be associated with intestinal epithelial shedding due to gut dysbiosis^38^. This increase could reflect a compensatory response to oxidative stress or inflammation, both of which are common in IBS^39^. While further research is needed to determine its diagnostic significance, these findings suggest that disturbances in serine metabolism may contribute to IBS pathophysiology and warrant further investigation. To further explore sex-specific metabolic differences, we performed a stratified analysis by sex. In the female subgroup, no urinary metabolites were significantly different between healthy controls and IBS patients. In the male subgroup, exploratory analysis indicated differences in several metabolites (**Fig.****S1**). Specifically, galactose and mannose tended to be higher in IBS patients, whereas 2-oxoglutaric acid, ribose, and threonic acid appeared higher in healthy controls (*p* < 0.05). These findings may reflect underlying metabolic disruptions that differ by sex. The lower urinary 2-oxoglutaric acid observed in male IBS-D patients could indicate impaired TCA cycle activity and reduced energy production in intestinal epithelial cells. This is supported by prior studies showing sex-based differences in urinary TCA intermediates, with males typically exhibiting higher baseline levels of 2-oxoglutaric acid in healthy states^40^. Thus, the relative decrease of 2-oxoglutaric acid in our IBS cohort may represent a sex-specific response to intestinal stress or dysbiosis. Similarly, the elevated levels of galactose and mannose in male IBS patients may suggest altered carbohydrate metabolism or malabsorption. Although limited, evidence from Nybacka et al.^41^ showed that dietary interventions in IBS can affect urinary excretion of sugar alcohols and monosaccharides, possibly via modulation of gut microbial activity or epithelial transport. This implies that male IBS-D patients may exhibit a distinct metabolic adaptation to dysregulated carbohydrate processing. While these results are intriguing, it should be noted that recent dietary intake and medication use, including antibiotics, probiotics, and low-FODMAP adherence, were not systematically controlled in this study. Such factors are known to influence both microbiota composition and urinary sugar profiles and may have contributed to the observed differences. Nevertheless, the consistent elevation of fructose, mannose, and galactose across analyses highlights their potential relevance to IBS pathophysiology, warranting cautious interpretation and validation in future studies. In this context, the observed sex-related differences further underscore the importance of considering both biological sex and lifestyle factors in biomarker discovery and mechanistic investigations.

Distinct metabolic compositions between groups were further examined using PCoA based on Bray-Curtis and Jaccard indices (**Fig. S2**). As with the gut microbiota analysis, the beta diversity analysis of the urinary metabolites did not reveal a statistically significant difference between the healthy and IBS groups. However, the relatively lower *p*-values in the urinary metabolites analysis suggest that urinary metabolites may be more sensitive than gut microbial composition in detecting metabolic disturbances associated with IBS. Keshteli et al.^40^ found that IBS patients have a distinct urinary metabolomic profile with altered amino acids, phosphatidylcholine, and dopamine levels, enabling differentiation from ulcerative colitis and healthy individuals. Since metabolic changes can reflect both host physiological responses and gut microbiota activity, urine-based metabolic profiling could serve as a potentially valuable approach for IBS characterization, complementing gut microbiota analysis^41^.

**Fig. 4:**
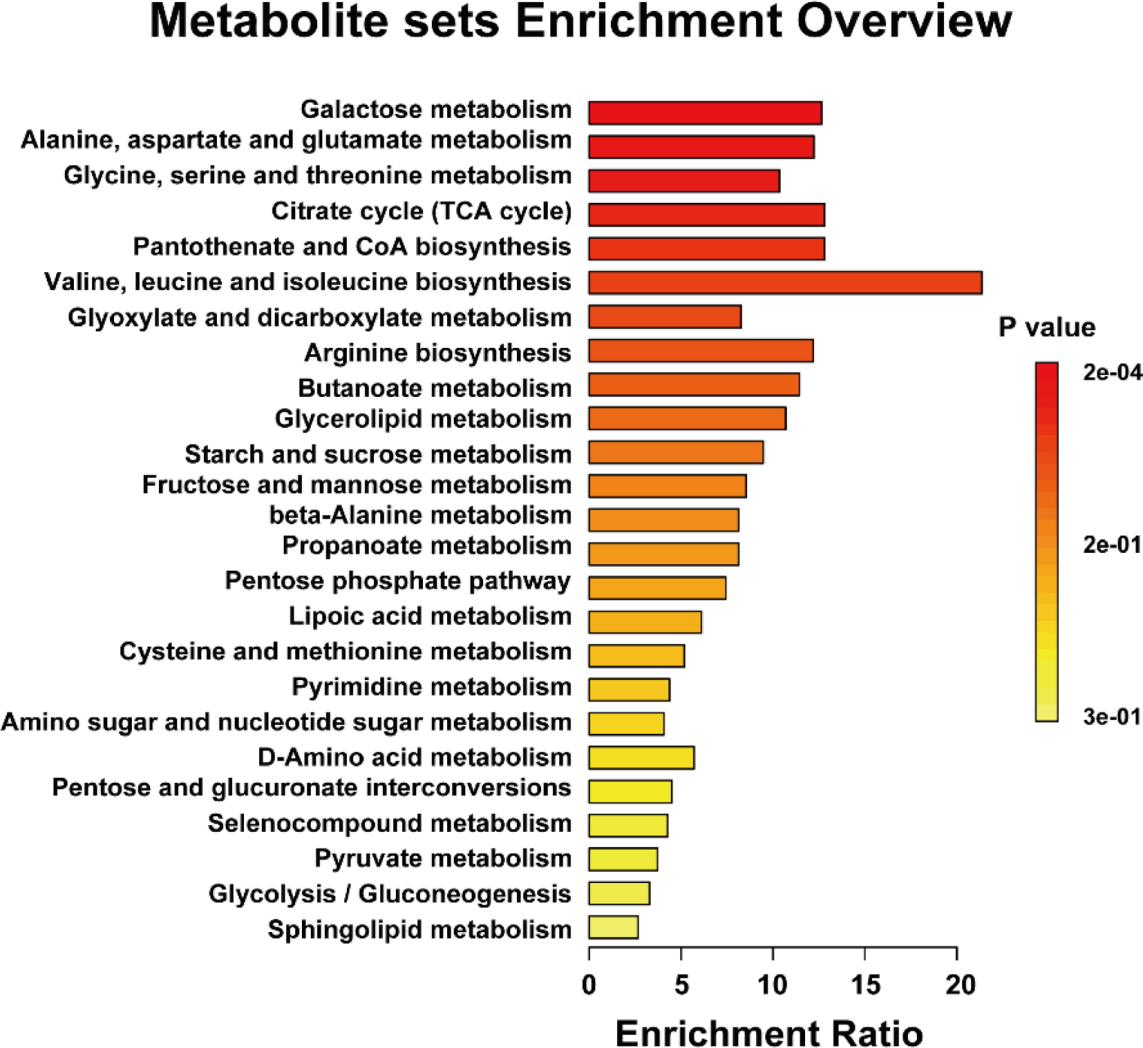
MSEA conducted in MetaboAnalyst using urinary metabolite profiles from the healthy and IBS groups. Metabolite set enrichment overview, where the enrichment ratio represents the proportion of detected metabolites within each pathway. Colors indicate statistical significance with darker red representing lower *p*-values.

To further elucidate the metabolic alterations associated with IBS, MSEA was conducted (Fig. 4). The results revealed significant enrichment of pathways related to galactose metabolism, amino acid metabolism - including alanine, aspartate, glutamate, glycine, serine, and threonine - as well as the TCA cycle, indicating potential metabolic dysregulation. Disruptions in galactose metabolism may lead to malabsorption, increasing luminal osmolarity and promoting microbial fermentation. This produces gas and SCFAs, contributes to bloating, diarrhea, and gut discomfort in IBS patients^42^. Additionally, impairments in the TCA cycle, a key pathway for cellular energy production, may reduce ATP availability in intestinal epithelial cells, weakening gut barrier integrity and exacerbating inflammation^43^. These metabolic disturbances highlight potential mechanisms contributing to IBS symptoms and warrant further investigation. In line with these pathway enrichments, threonine, mannose, galactose, and urea exhibited high VIP scores in this study, further emphasizing their relevance to IBS-related metabolic disturbances. Their involvement in carbohydrate and amino acid metabolism suggests that these compounds may contribute to the broader biochemical dysregulation observed in IBS patients. Additionally, previous multi-omics studies have highlighted disturbances in carbohydrate and amino acid metabolism in IBS patients, reinforcing the relevance of these findings^44^.

Recent studies have also shown that gut microbiota-derived metabolites, including SCFAs and amino acid derivatives, play a role in IBS pathophysiology by influencing gut motility and immune responses^45^. These results indicate that metabolic disturbances in IBS extend beyond individual metabolites, affecting broader biochemical pathways involved in energy production, amino acid utilization, and carbohydrate metabolism. The enrichment of these pathways suggests that metabolic dysregulation may be a key factor contributing to symptom manifestation in IBS, potentially influencing gut motility, inflammation, and microbial interactions.

### Predictive performance of urinary metabolites and gut microbiota for IBS classification

**Fig. 5:**
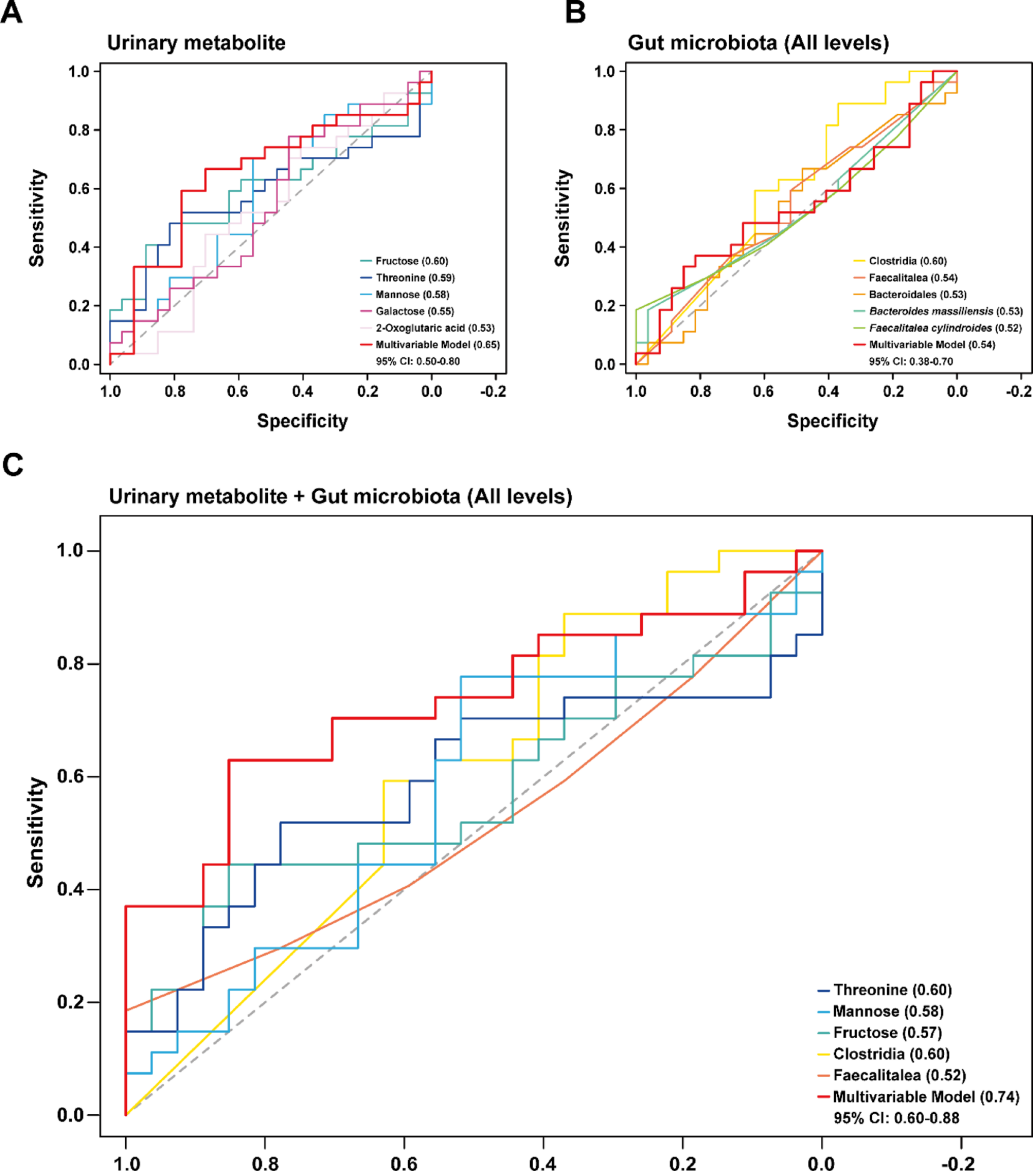
ROC curve analyses of the identified urinary metabolites and gut microbiota features for IBS prediction. ROC curve analyses of (A) selected urinary metabolites features and (B) gut microbiota taxonomic features and (C) a combined urinary metabolites and gut microbiota model in distinguishing between the healthy and IBS groups. Each curve represents the predictive performance of individual biomarkers or mixed models. The area under the curve (AUC) values and optimal cutoffs reflect the ability of the urine metabolites and gut microbiota features to discriminate between groups. The AUC values for each feature are indicated in parentheses following the respective feature names. The dashed line represents the random classification boundary (AUC = 0.5).

To evaluate the predictive potential of urinary metabolites and gut microbiota in distinguishing healthy individuals from those with IBS, ROC curve analyses were performed as shown in Fig. 5. First, the predictive performance of selected urinary metabolites was assessed (Fig. 5A). The identified metabolites exhibited varying degrees of predictive power, as reflected in their AUC values. Among them, fructose (AUC = 0.60), threonine (AUC = 0.59), and mannose (AUC = 0.58) demonstrated the highest predictive accuracy, suggesting their potential as biomarkers for IBS classification. To further enhance predictive performance, a logistic regression model incorporating all selected urinary metabolites was developed. The multivariable model achieved an AUC of 0.65, outperforming individual metabolites, indicating that a combined approach enhances IBS classification accuracy based on urinary metabolic signatures. Next, the diagnostic capacity of gut microbiota taxonomic features was evaluated across all levels (Fig. 5B). While individual microbial taxa, such as Clostridia (AUC = 0.60) and *Faecalitalea* (AUC = 0.54), displayed moderate discriminatory power, the overall performance of the gut microbiota-based model was lower than that of the urine metabolite-based model with a multivariable model AUC of 0.54. To determine whether integrating urinary metabolites and gut microbiota would enhance IBS prediction, a combined model was constructed as shown in Fig. 5C. The multivariable model, incorporating both metabolomic and gut microbiota features, yielded an AUC of 0.74, demonstrating the highest classification performance. This result highlights the advantage of a multi-marker approach, where integrating metabolic and microbial biomarkers provides a more comprehensive assessment of IBS-associated alterations. While urinary metabolites demonstrate stronger predictive potential than gut microbiota, combining both datasets further enhances classification accuracy of IBS, reinforcing the value of an integrated approach. As all IBS participants in this study were classified as IBS-D, the ROC analysis was conducted specifically for this subtype and thus does not extend to other IBS subtypes such as IBS-C or IBS-M. In addition, while the multivariable models demonstrated favorable classification performance, external validation was not conducted in this study. As a result, the AUC values may reflect optimistic estimates based on the discovery dataset. Future research including independent validation cohorts will be essential to confirm the robustness and generalizability of these predictive models. Future studies with larger, independent cohorts will allow the implementation of more comprehensive approaches, including bootstrap-based optimism correction, coefficient stability assessment, and the evaluation of sparser models. To further enhance reproducibility, subsequent work should also incorporate a pre-registered validation plan specifying model construction, feature selection, and evaluation procedures in advance, as well as advanced validation frameworks such as nested cross-validation and calibration analyses (e.g., calibration slope, Brier score). Collectively, these measures will minimize bias and ensure a more transparent and reliable assessment of predictive performance.

Despite extensive metabolomic research on IBS, most studies have focused on fecal and serum metabolites, while relatively few have explored urinary metabolites. As urine reflects systemic metabolic changes and microbial interactions, its diagnostic potential in IBS remains unexamined. To the best of our knowledge, this study is the first to present a multivariable model for IBS prediction by integrating urine metabolomics with gut microbiota profiling. By combining these two datasets, this research provides new insights into IBS pathophysiology and highlights the potential for a more comprehensive diagnostic approach. However, IBS heterogeneity and multifactorial influences, including diet and genetics, necessitate further studies with strain-level microbiota analysis, functional profiling, and larger cohorts to refine IBS-specific microbial and metabolic signatures. Although this study did not implement formal exclusion criteria or control protocols related to recent dietary intake or medication use (e.g., antibiotics, probiotics, or low-FODMAP diets), such factors are known to influence gut microbiota composition and urinary metabolite profiles, particularly monosaccharides like fructose. The observed elevation of urinary fructose in IBS patients is consistent with prior reports of impaired carbohydrate absorption; however, given the dietary sensitivity of this metabolite, its diagnostic relevance should be interpreted cautiously. Future studies incorporating standardized assessments of dietary habits and medication histories will be essential to validate these findings and clarify the specificity of urinary fructose as a potential biomarker. In addition, as the current cohort includes only IBS-D patients, future studies incorporating diverse IBS subtypes will be essential to assess the generalizability and subtype-specific performance of predictive models. Furthermore, although sex-stratified analyses were conducted, the imbalance in sex distribution, particularly the higher proportion of females in the control group, may have influenced group comparisons of both microbiota and metabolite profiles. Future studies with larger, sex-balanced cohorts are warranted to validate and strengthen these findings.

## Conclusions

This study explored the diagnostic potential of urine metabolomics and gut microbiota profiling in distinguishing IBS patients from healthy individuals. Although no significant differences in gut microbiota diversity were observed, IBS patients exhibited distinct microbial signatures, including increased levels of Bacteroidales and *Faecalitaliea*. Urinary metabolite analysis showed significantly elevated fructose levels in IBS patients with trends of increased serine, mannose, and galactose, indicating metabolic alterations. While ROC curve analysis indicated that urinary metabolites had stronger discriminatory power than gut microbiota, and that combining both datasets improved classification performance, these findings were derived solely from within-sample cross-validation and lack external validation. Therefore, the results should be interpreted as preliminary and hypothesis-generating rather than confirmatory. The findings highlight the potential utility of integrative omics approaches in IBS research. However, validation in larger, sex-balanced, and clinically diverse cohorts is essential to confirm these associations and to assess their diagnostic applicability.

## Supplementary Information

Below is the link to the electronic supplementary material.

Supplementary Material 1

## Data Availability

The datasets generated and analyzed in the current study are available in the NCBI Sequence Read Archive (SRA) under the Bioproject accession number PRJNA1242794 (https://www.ncbi.nlm.nih.gov/bioproject/PRJNA1242794). Raw GC-MS data of urinary metabolites are accessible through MetaboLights under accession ID [MTBLS12737](https:/www.ebi.ac.uk/metabolights/editor/MTBLS12737/descriptors) ([https://www.ebi.ac.uk/metabolights/MTBLS12737](https:/www.ebi.ac.uk/metabolights/MTBLS12737)). Additional data supporting the findings of this study, including full feature matrices, processing parameters (MS-DIAL, identification scores, RI windows), and reporting checklists (STORMS for microbiome and MSI for metabolomics), are provided in the Supplementary Information.

## Abbreviations

AUC: Area under the curve
GC-MS: Gas chromatography-mass spectrometry
IBS: Irritable bowel syndrome
LEfSe: Linear discriminant analysis effect size
MSEA: Metabolite set enrichment analysis
PCoA: Principal coordinate analysis
PERMANOVA: Permutational multivariate analysis of variance
ROC: Receiver operating characteristic
16S rRNA: 16S ribosomal RNA
