# Supplementary material for: Prediction of irritable bowel syndrome by integrating urine metabolites and gut microbiota
Source: Sci Rep. 2025 Oct 31;15:38246. doi: 10.1038/s41598-025-22068-1 (PMC12578838; doi:10.1038/s41598-025-22068-1)

**Supplementary information**

**Table S1.** IBS symptom assessment questionnaire used to evaluate abdominal symptoms and bowel habit disturbances.

| IBS Symptom Assessment Questionnaire  Patient Name: __________  Patient ID: __________  Date: __________  Instructions: This questionnaire is designed to assess abdominal symptoms, bowel habits, and support Rome IV-based IBS subtype classification. Please answer the following questions based on your symptoms over the past 10 days. Tick the appropriate boxes or write your response where indicated. |
| --- |

| **No.** | **Item** | | | **Response Format** |
| --- | --- | --- | --- | --- |
| **Q1** | Have you experienced abdominal pain in the past 10 days? | | | □ Yes  □ No |
| **Q2** | If yes, how severe was the abdominal pain? (0–100 scale) | | | Score: _______ / 100 |
| **Q3** | On how many of the past 10 days did you experience abdominal pain? | | | Days: ______ / 10 |
| **Q4** | Have you experienced abdominal bloating in the past 10 days? | | | □ Yes  □ No |
| **Q5** | If yes, how severe was the bloating? (0–100 scale) | | | Score: _______ / 100 |
| **Q6** | How would you describe your current bowel habits? | | | ________________ |
| **Q7** | Do your bowel habits interfere with your daily life? | | | □ Yes  □ No |
| **Q8** | During the past 7 days, was the average intensity of your most severe abdominal pain 3.0 or higher (on a 0–10 scale)? | | | □ Yes  □ No |
| **Q9** | During the past 7 days, on how many days did you have at least one stool classified as Bristol type 6 or 7? | | | Days: ______ / 7 |
| **Q10** | Please record your bowel movements for the past 7 days. Include number of bowel movements and Bristol stool type(s) per day. | | |  |
|  | Day | **10-1**  No. of Bowel Movements | **10-2**  Bristol Stool Type(s) |  |
|  | 1 |  |  |  |
|  | 2 |  |  |  |
|  | 3 |  |  |  |
|  | 4 |  |  |  |
|  | 5 |  |  |  |
|  | 6 |  |  |  |
|  | 7 |  |  |  |

|  | | Healthy (n=27) | | IBS (n=27) | *p*-value |
| --- | --- | --- | --- | --- | --- |
| Age (range) | 44.00 ±9.03 (27-62) | | 43.56 ±9.89 (21-62) | | 0.865 |
| Sex (Male / Female) | 3 (11.11%) / 24 (88.89%) | | 14 (51.85%) / 13 (48.15%) | | < 0.001 |
| BMI | 23.74 ± 2.74 | | 22.42 ± 3.61 | | 0.136 |

**Table S2.** Characteristics of participants in the healthy and IBS groups.

**Table S3**. STORMS (Strengthening The Organization and Reporting of Microbiome Studies) checklist completed for this study. Each item is marked as Yes, No, or NA depending on whether it was addressed in the manuscript, with corresponding comments indicating the section, figure, or table where the information can be found.

| **Number** | **Item** | **Yes/No/NA** | **Comments or location in manuscript** |
| --- | --- | --- | --- |
| **Abstract** | | | |
| 1.0 | Structured or Unstructured Abstract | Yes | Abstract |
| 1.1 | Study Design | Yes | Abstract |
| 1.2 | Sequencing methods | Yes | Abstract |
| 1.3 | Specimens | Yes | Abstract |
| **Introduction** | | | |
| 2.0 | Background and Rationale | Yes | Background |
| 2.1 | Hypotheses | Yes | Background |
| **Methods** | | | |
| 3.0 | Study Design | Yes | Materials & methods 2.1 |
| 3.1 | Participants | Yes | Materials & methods 2.1 |
| 3.2 | Geographic location | Yes | Materials & methods 2.1 |
| 3.3 | Relevant Dates | No | Materials & methods 2.1 |
| 3.4 | Eligibility criteria | Yes | Materials & methods 2.1 |
| 3.5 | Antibiotics Usage | No | Results and discussion 3.4, limitation noted). |
| 3.6 | Analytic sample size | Yes | Materials & methods 2.1 (n=54 total; Table S2 shows demographics) |
| 3.7 | Longitudinal Studies | NA | Not a longitudinal study. |
| 3.8 | Matching | NA | No matching design. |
| 3.9 | Ethics | Yes | Institutional review board approval (NJ-IRB-23-1) and informed consent obtained. |
| 4.0 | Laboratory methods | Yes | Materials & methods 2.3–2.4: sequencing and metabolomics labs described. |
| 4.1 | Specimen collection | Yes | Materials & methods 2.2: fecal and urine collection protocols. |
| 4.2 | Shipping | Yes | Samples transported on ice packs to laboratory. |
| 4.3 | Storage | Yes | Samples stored at −80 °C until analysis. |
| 4.4 | DNA extraction | Yes | Methods 2.3: DNA extraction kit/protocol. |
| 4.5 | Human DNA sequence depletion or microbial DNA enrichment | NA | Not applicable for 16S rRNA amplicon sequencing. |
| 4.6 | Primer selection | Yes | Materials & methods 2.3 (515F/806R primers targeting V4 region) |
| 4.7 | Positive Controls | No | Not reported. |
| 4.8 | Negative Controls | No | Not reported. |
| 4.9 | Contaminant mitigation and identification | No | Not reported. |
| 4.10 | Replication | No | No biological or technical replicates mentioned. |
| 4.11 | Sequencing strategy | Yes | 16S rRNA amplicon sequencing on Illumina MiSeq platform. |
| 4.12 | Sequencing methods | Yes | Average read counts and processing pipeline described (Methods 2.3). |
| 4.13 | Batch effects | No | Batch randomization or correction not described. |
| 4.14 | Metatranscriptomics | NA | Not performed. |
| 4.15 | Metaproteomics | NA | Not performed |
| 4.16 | Metabolomics | Yes | Methods 2.4: GC-MS workflow, derivatization, RTX-5MS column, ribitol internal standard, QC samples every 30 injections |
| 5.0 | Data sources/  measurement | Yes | Materials & methods 2.1 |
| 6.0 | Research design for causal inference | Yes | \| Potential confounding (diet, sex imbalance) acknowledged in Section 3.4  and Results and discussion. \| \| --- \| |
| 6.1 | Selection bias | No | Not explicitly discussed. |
| 7.0 | Transformations / normalization | Yes | Materials and methods 2.5 |
| 7.1 | Quality Control | Yes | Materials and methods 2.3 |
| 7.2 | Sequence analysis | Yes | Materials and methods 2.3 |
| 7.3 | Statistical methods | Yes | Materials and methods 2.5 |
| 7.4 | Longitudinal analysis | NA | Not applicable. |
| 7.5 | Subgroup analysis | Yes | Results and discussion 3.3 |
| 7.6 | Missing data | No | Missing data handling not described. |
| 7.7 | Sensitivity analyses | No | Not performed; leave-one-sex/site-out suggested as future plan in Discussion. |
| 7.8 | Findings | Yes | Materials and methods 2.5 |
| 7.9 | Software | Yes | Materials and methods 2.3, 2.5 |
| 8.0 | Reproducible research | Yes | Not applicable. |
| 8.1 | Raw data access | Yes | Data availability (16S: SRA PRJNA1242794)   \|  \| \| --- \| |
| 8.2 | Processed data access | Yes | Supplementary Tables S6 |
| 8.3 | Participant data access | No | Individual-level mapping not provided |
| 8.4 | Source code access | No | Analysis scripts not deposited. |
| 8.5 | Full results | Yes | Supplementary Tables S6 |
| **Results** | | | |
| 9.0 | Descriptive data | Yes | Supplementary Tables S2 |
| 10.0 | Microbiome data | Yes | Results and discussion 3.2 |
| 10.1 | Taxonomy | Yes | Results and discussion 3.2–3.3 |
| 10.2 | Differential abundance | Yes | Results and discussion 3.3 |
| 10.3 | Other data types | Yes | Urinary metabolomics (Results and discussion 3.4, Figs. 3–4, Table S7). |
| 10.4 | Other statistical analysis | Yes | Results and discussion 3.2, 3.5 |
| **Discussion** | | | |
| 11.0 | Key results | Yes | Results and discussion |
| 12.0 | Interpretation | Yes | Results and discussion, Conclusion |
| 13.0 | Limitations | Yes | Results and discussion |
| 13.1 | Bias | Yes | Results and discussion (Sex imbalance, uncontrolled diet/medications discussed) |
| 13.2 | Generalizability | Yes | Conclusion |
| 14.0 | Ongoing/future work | Yes | Results and discussion 3.5 |
| **Other information** | | | |
| 15.0 | Funding | Yes | Funding |
| 15.1 | Acknowledgements | Yes | Acknowledgements |
| 15.2 | Conflicts of Interest | Yes | Competing interests |
| 16.0 | Supplements | Yes | Electronic supplementary material |
| 17.0 | Supplementary data | Yes | Supplementary Tables |

**Table S4**. MSI (Metabolomics Standards Initiative) checklist completed for this study. Each item is marked as Yes, No, or NA depending on whether it was addressed in the manuscript, with corresponding locations indicating the section, figure, or table where the information can be found.

| **Number** | **Item** | **Yes/No/NA** | **Location in manuscript** |
| --- | --- | --- | --- |
| **ECWSG (Experimental Conditions Working Group)** | | | |
| 1.0 | Study design clearly stated | Yes | Materials and methods 2.1 (Study cohort) |
| 1.1 | Sample type and source  (e.g., urine, feces) | Yes | Materials and methods 2.2 (Sample collection) |
| 1.2 | Sample collection protocol | Yes | Materials and methods 2.2 |
| 1.3 | Sample transport and storage conditions | Yes | Materials and methods 2.2 (transport in cooler bags, storage at –80 °C) |
| 1.4 | Ethical approval and informed consent | Yes | Materials and methods 2.1 (Ethics approval, NJ-IRB-23-1) |
| 1.5 | Exclusion/inclusion criteria | Yes | Materials and methods 2.1 (Eligibility criteria) |
| 1.6 | Replicates (biological/technical) | Yes | QC samples described, Materials and methods 2.4 |
| 1.7 | Quality control samples | Yes | Materials and methods 2.4 (pooled QC, blanks) |
| 1.8 | Randomization/batch effects accounted | Yes | Materials and methods 2.4 (QC/blank injection order) |
| **DA (Data Analysis Standards)** | | | |
| 2.0 | Instrument type and settings | Yes | Materials and methods 2.4 (GC–MS QP2020 NX, RTX-5MS, temperature program, etc.) |
| 2.1 | Derivatization and sample prep details | Yes | Materials and methods 2.4 (methoximation, silylation, reagents listed) |
| 2.2 | Internal standards used | Yes | Materials and methods 2.4 (ribitol, 0.5 mg/mL) |
| 2.3 | Data pre-processing software | Yes | Materials and methods 2.4 (Shimadzu Postrun Analysis, MS-DIAL v.4.9) |
| 2.4 | Metabolite identification criteria | Yes | Materials and methods 2.4 (RI ±20, EI similarity ≥90%, ID score ≥90%, NIST 20.0) |
| 2.5 | Data normalization procedures | Yes | Materials and methods 2.4 (ribitol, TPA normalization) |
| 2.6 | Statistical analysis methods | Yes | Materials and methods 2.5 (Welch’s t-test, Mann–Whitney, PERMANOVA, ROC, etc.) |
| 2.7 | Multiple testing correction | Yes | Materials and methods 2.5 (Benjamini–Hochberg FDR) |
| 2.8 | Software and versions | Yes | Materials and methods 2.5 (R v4.3.3, SIMCA-P v17.0, GraphPad Prism v9.4.1, MetaboAnalyst 6.0) |
| 2.9 | Data availability (raw & processed) | Yes | Data availability section (MTBLS12737, Supplementary) |

**Table S5.** Abdominal symptom severity and Rome IV subtype classification in the IBS group (n = 27).

| Parameter | Mean ±SD | Subtype |
| --- | --- | --- |
| Q2. Abdominal pain severity score (0–100 scale) | 53.96 ±12.34 | IBS-D |
| Q3. Number of pain days out of 10 | 4.07 ±1.57 |  |
| Q5. Abdominal bloating severity score (0–100 scale) | 50.75 ±16.69 |  |
| Q8. Proportion with abdominal pain ≥3.0 (0–10 scale) | 100% (27/27) |  |
| Q9. Number of days (out of 7) with at least one stool classified as Bristol type 6 or 7 | 3.85 ±1.51 |  |

**Table S6.** Summary of statistical testing results for gut microbial taxa at the genus level between the Healthy and IBS groups. The table presents raw *p*-values (Welch’s t-test or Mann–Whitney U test), FDR-adjusted *q*-values, and effect sizes (Hedges’ *g*).

| **No.** | **Genus** | ***p*-value** | ***q*-value** | **Hedges' *g*** |
| --- | --- | --- | --- | --- |
| 1 | *Bacteroides* | 0.8503 | 0.9302 | 0.1369 |
| 2 | *Prevotella* | 0.8638 | 0.9302 | 0.0955 |
| 3 | *Faecalibacterium* | 0.2753 | 0.6424 | 0.2980 |
| 4 | *Sutterella* | 0.7967 | 0.9302 | -0.1511 |
| 5 | *Parabacteroides* | 0.7444 | 0.9302 | 0.2903 |
| 6 | *Megamonas* | 0.1971 | 0.6424 | -0.1239 |
| 7 | *Phascolarctobacterium* | 0.6808 | 0.9302 | 0.1729 |
| 8 | *Subdoligranulum* | 0.4020 | 0.7511 | -0.0640 |
| 9 | Escherichia-Shigella | 0.9589 | 0.9589 | 0.0016 |
| 10 | *Lachnospira* | 0.4022 | 0.7511 | -0.1286 |
| 11 | *Alistipes* | 0.2395 | 0.6424 | -0.3057 |
| 12 | *Dialister* | 0.1164 | 0.6424 | -0.1398 |
| 13 | *Agathobacter* | 0.2211 | 0.6424 | 0.6504 |
| 14 | *Veillonella* | 0.8859 | 0.9302 | 0.0718 |
| 15 | UCG-002 | 0.2722 | 0.6424 | -0.4003 |
| 16 | *Bifidobacterium* | 0.1858 | 0.6424 | 0.2417 |
| 17 | *Roseburia* | 0.6554 | 0.9302 | -0.2147 |
| 18 | *Lachnoclostridium* | 0.4292 | 0.7511 | 0.0266 |
| 19 | Christensenellaceae  R-7 group | 0.1455 | 0.6424 | -0.1783 |
| 20 | Eubacterium coprostanoligenes group | 0.2671 | 0.6424 | -0.4123 |
| 21 | *Blautia* | 0.7969 | 0.9302 | 0.1425 |

**Table S7.** Summary of statistical testing results for urinary metabolites between the Healthy and IBS groups. The table presents raw p-values (Welch’s t-test or Mann–Whitney U test), FDR-adjusted q-values, and effect sizes (Hedges’ *g*)

| **No.** | **Metabolite** | ***p*-value** | ***q*-value** | **Hedges' *g*** |
| --- | --- | --- | --- | --- |
| 1 | 2-Hydroxypyridine | 0.4784 | 0.8392 | 0.1915 |
| 2 | 2-Oxoglutaric acid | 0.3184 | 0.8250 | -0.3870 |
| 3 | 3-Hydroxyisovaleric acid | 0.6065 | 0.8392 | -0.0588 |
| 4 | Alanine | 0.3442 | 0.8250 | 0.3397 |
| 5 | Benzoic acid | 0.8874 | 0.9761 | -0.1687 |
| 6 | β -Alanine | 0.6216 | 0.8392 | 0.2829 |
| 7 | Fructose | 0.0326 | 0.8250 | 0.4877 |
| 8 | Galactose | 0.2782 | 0.8250 | 0.4067 |
| 9 | Glyceric acid | 0.8404 | 0.9761 | 0.2590 |
| 10 | Glycerol | 0.1243 | 0.8250 | -0.2406 |
| 11 | Glycolic acid | 0.3713 | 0.8250 | -0.0196 |
| 12 | Hydroxylamine | 0.1424 | 0.8250 | 0.3256 |
| 13 | Lactic acid | 0.5478 | 0.8392 | -0.2529 |
| 14 | Maltose | 0.9761 | 0.9761 | 0.0607 |
| 15 | Mannose | 0.1902 | 0.8250 | 0.4324 |
| 16 | Phenol | 0.9590 | 0.9761 | -0.3232 |
| 17 | Phosphate | 0.5521 | 0.8392 | -0.1605 |
| 18 | Pyruvic acid | 0.4920 | 0.8392 | -0.1924 |
| 19 | Ribose | 0.6040 | 0.8392 | -0.1400 |
| 20 | Serine | 0.3101 | 0.8250 | 0.3959 |
| 21 | Succinic acid | 0.9044 | 0.9761 | -0.0259 |
| 22 | Threonic acid | 0.9502 | 0.9761 | 0.0168 |
| 23 | Threonine | 0.1624 | 0.8250 | 0.5289 |
| 24 | Uracil | 0.2706 | 0.8250 | -0.2073 |
| 25 | Urea | 0.1348 | 0.8250 | -0.4077 |
| 26 | Valine | 0.3972 | 0.8250 | 0.2941 |
| 27 | Xylose | 0.6836 | 0.8789 | 0.2527 |

**Supplementary README Note**

**1. Overview**

This README note provides detailed information on supplementary datasets, data availability, processing parameters, and reporting checklists associated with the manuscript. It is designed to ensure transparency, reproducibility, and compliance with community standards, including STORMS (microbiome) and MSI (metabolomics).

**2. Data Availability**

- Microbiome (16S rRNA sequencing): Raw sequencing data are deposited in the NCBI Sequence Read Archive (SRA) under Bioproject accession PRJNA1242794.
- Metabolomics (GC–MS, urine): Raw metabolomics files are available at MetaboLights, accession MTBLS12737.
- Supplementary materials: Supplementary tables and figures, as well as reporting checklists, are provided within this document.

**3. Supplementary File Contents**

- **Table S1.** IBS symptom severity questionnaire scores
- **Table S2.** Baseline demographics of participants
- **Table S3.** STORMS checklist for microbiome reporting
- **Table S4.** MSI checklist for metabolomics reporting
- **Table S5.** Clinical characterization of IBS patients
- **Table S6.** Raw *p*-values, FDR-adjusted *q*-values, and effect sizes (Hedges’ *g*) for microbial taxa
- **Table S7.** Raw *p*-values, FDR-adjusted *q*-values, and effect sizes (Hedges’ *g*) for urinary metabolites
- **Figures S1–S2.** Supplementary visualizations (sex-stratified metabolite profiles, β-diversity plots)

**4. Processing Parameters**

**Microbiome (16S rRNA sequencing)**

- Platform: Illumina MiSeq, paired-end 2 × 250 bp
- Region: V4 (primers 515F/806R)
- Pipeline: QIIME2 framework
- Denoising/ASV inference: DADA2 v1.16
- Taxonomic assignment: Naïve Bayes classifier trained on SILVA 138.1
- Average read depth: Mean 116,812 (range 44,508–150,242)
- Quality control: Negative controls included; ASV filtering and chimera removal applied

**Metabolomics (GC–MS urine analysis)**

- Instrument: Shimadzu GCMS-QP2020 NX with RTX-5MS capillary column
- Derivatization: Methoximation with O-methoxyamine hydrochloride, followed by MSTFA silylation
- Internal standard: Ribitol (0.5 mg/mL)
- QC strategy: Pooled QC samples and blanks injected at start and every 20 samples
- Identification criteria: RI tolerance ±20, EI similarity ≥90%, identification score ≥90%, confirmed with NIST v20.0 and authentic standards
- Data processing: Shimadzu Postrun Analysis → ABF → MS-DIAL v4.9
- Normalization: Peak intensities normalized to ribitol, then to total peak area (TPA)
- Statistical analysis: Welch’s *t*-test, Mann–Whitney U test, Kruskal–Wallis, PERMANOVA, FDR correction, ROC modeling with ridge logistic regression

**5. Reporting Standards**

- STORMS (microbiome): Checklist completed and provided (Table S3).
- MSI (metabolomics): Checklist completed and provided (Table S4).

**6. Notes**

This README note should be cited together with the manuscript and supplementary materials to ensure reproducibility. Future reuse of the data should reference both the NCBI SRA and MetaboLights accession numbers.

**Fig. S1.** Box plots comparing the peak intensity of urine metabolites (*p*-values < 0.05) between the healthy and IBS groups in male subjects. Data are shown as box plots with whiskers representing mean ± SD. *P* values for comparisons were calculated via permutational analysis of variance. The 95% CIs of the mean values were: 2-Oxoglutaric acid (Healthy: 0.000098–0.007189; IBS: 0.00116–0.002340), Galactose (Healthy: 0.003199–0.006419; IBS: –0.003148–0.01663), Mannose (Healthy: 0.05355–0.1126; IBS: –0.02414–0.2192), Ribose (Healthy: 0.006171–0.01031; IBS: 0.004606–0.007167), and Threonic acid (Healthy: 0.05287–0.1351; IBS: 0.03935–0.07052).


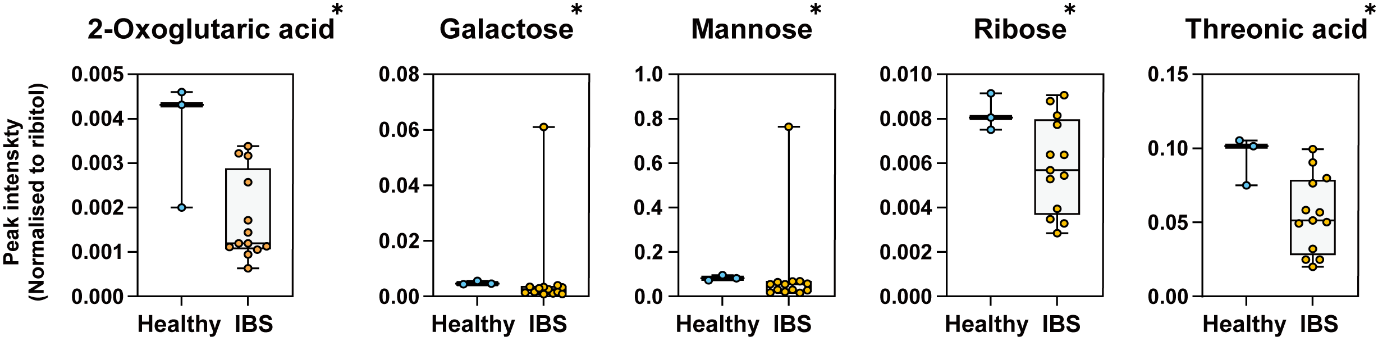


**Fig. S2.** PCoA of urinary metabolites in healthy and IBS groups, based on Bray-Curtis and Jaccard indices.


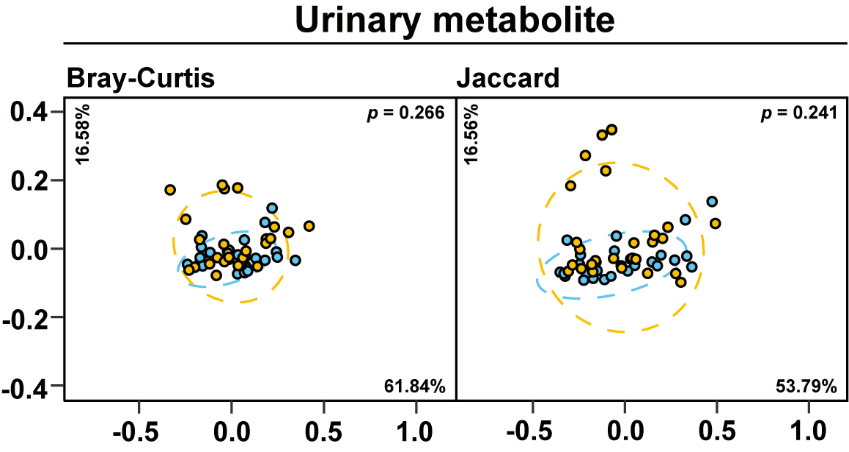

Supplement: Supplementary file 1 — Supplementary Material 1 [file 41598_2025_22068_MOESM1_ESM.docx]
